# First Detection of *Francisella halioticida* Infecting a Wild Population of Blue Mussels *Mytilus edulis* in the United Kingdom

**DOI:** 10.3390/pathogens11030329

**Published:** 2022-03-08

**Authors:** Irene Cano, Abigail Parker, Georgia M. Ward, Matthew Green, Stuart Ross, John Bignell, Caroline Daumich, Rose Kerr, Stephen W. Feist, Frederico M. Batista

**Affiliations:** 1 Cefas Weymouth Laboratory, International Centre of Excellence for Aquatic Animal Health, Barrack Road, Weymouth DT4 8UB, UK; abigail.parker@cefas.co.uk (A.P.); georgia.m.ward@cefas.co.uk (G.M.W.); matthew.green@cefas.co.uk (M.G.); stuart.ross@cefas.co.uk (S.R.); john.bignell@cefas.co.uk (J.B.); caroline.daumich@cefas.co.uk (C.D.); rose.kerr@cefas.co.uk (R.K.); oie.cceaad@cefas.co.uk (S.W.F.); frederico.batista@cefas.co.uk (F.M.B.); 2 Department of Life Sciences, The Natural History Museum, Cromwell Road, London SW7 5BD, UK

**Keywords:** *Francisella halioticida*, blue mussels *Mytilus edulis*, prokaryote cyst, granulocytoma, intracellular bacterium, Nanopore sequencing, 16S rRNA gene

## Abstract

In the last decade, declines in the population of wild blue mussels *Mytilus edulis* in the Tamar estuary (United Kingdom) have been noted. In archived samples collected from 2013 to 2019, between 7% (in 2013) and 18% (in 2019) showed large granulocytoma and haemocytic infiltration in the interstitial tissue of the digestive gland. Four samples were selected for *16S rRNA* gene Nanopore sequencing. A consensus sequence of 1449 bp showed nucleotide similarities between 99.93–100% with published sequences of *Francisella halioticida*. In situ hybridisation (ISH) confirmed the presence of *F. halioticida* DNA within individual granulocytes of granulocytomas and also in prokaryotic-like inclusion bodies within the digestive epithelial cells. The design of diagnostic tests for surveillance of *F. halioticida,* including more specific ISH probes and sequencing the genome of the isolates infecting mussels, will shed more light on the pathogenicity and spread of this pathogen.

## 1. Introduction

In the last decade, population declines have been sporadically noted in wild blue mussels (*Mytilus edulis* L.) in the Tamar estuary, in the southwest of the United Kingdom. Histology analysis conducted in 2011 showed the presence of intracellular microcolonies of bacteria (IMCs) in the digestive diverticula of blue mussels. Other pathogens and symbionts were also observed, such as gill ciliates (including *Ancistrum mytili*), the copepod *Mytilicola sp.*, Ciliophora-like organisms, and *Marteilia pararefringens* [1,2]. IMCs in the digestive gland were associated with host inflammation and the severity of the infection ranged from moderate to severe [3].

In an international effort to characterise IMCs infecting molluscs, a broad range of mollusc species was collected worldwide and subjected to *16S rRNA* gene sequencing [3]. Phylogenetic analysis of recovered Operational Taxonomic Units (OTUs) showed that endozoicomonadaceae sequences were dominant in most of the specimens analysed [3,4]. However, in samples from blue mussels collected in the Tamar River, the proportion of *Endozoicomonas*-like organisms was minimal (up to 0.3% of the reads). In its place, an unusually high proportion of reads (up to 67%) showed a high nucleotide sequence similarity with the *Francisella* genus, but identification to the species level was not possible. Furthermore, it was not investigated whether these *Francisella* reads were associated with the IMC lesions [3].

Among the members of the genus *Francisella*, the species *Francisella halioticida* emerged as a pathogen of molluscs in 2005 [5]. This intracellular bacterium was first reported in Japan, associated with mass mortality (84%) of the giant abalone (*Haliotis gigantea* Gmelin) [5]. The bacterium was isolated from infected hemolymph, and the isolate was used to confirm its pathogenicity by experimental infection, reaching cumulative mortality of 98.6% [5]. Phylogenetic analysis placed *F. halioticida* as a novel organism, with the establishment of the strain Shimane-1 as a new species within the genus, sharing a nucleotide identity of ca. 98% of the *16S rRNA* gene with isolates of *F. philomiragia* and *F. noatunensis* [6]. In 2015, *F. halioticida* was reported in Canada, associated with mortalities of Yesso scallops (*Mizuhopecten* (=*Patinopecten*) *yessoensis*) [7]. Two years later, *F. halioticida* was also associated with adductor muscle lesions in Yesso scallops in Japan [8]. Experimental infections with isolates from both the Canadian and Japanese outbreaks confirmed the high pathogenicity of *F. halioticida* to Yesso scallops, causing cumulative mortality rates of 100% by injection, and 60–86% by bath exposure [9]. In Europe, the presence of *F. halioticida* has been recently reported in France, infecting mussels *Mytilus* spp. associated with high mortality and population declines over the last years [10].

In the present study, we confirm for the first time the presence of *F. halioticida* infection in archived samples of blue mussels from the United Kingdom. We sequenced the almost complete *16S rRNA* gene of the bacterium using a novel Nanopore sequencing approach and confirmed its association with the bacterial lesions by in situ hybridization. The impact of this pathogen on wild population declines, and the risks of spreading to shellfish farms are discussed.

## 2. Results

### 2.1. Granulocytomas in Blue Mussels

**Figure 1:**
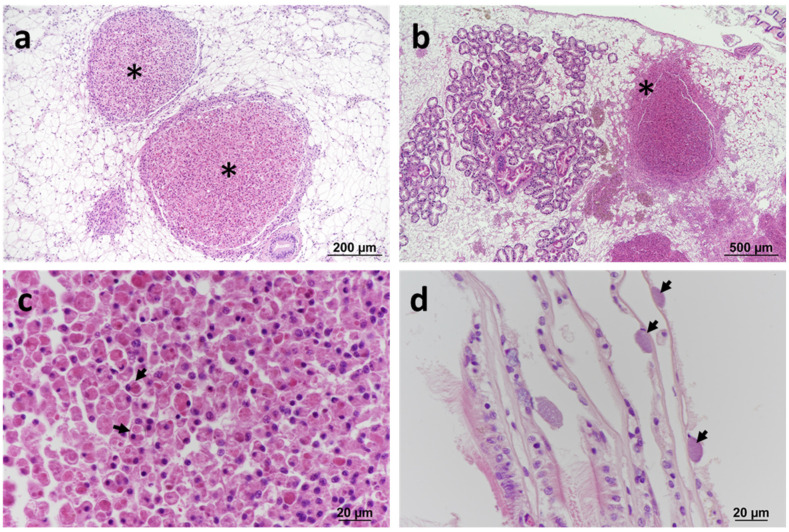
Histopathology of blue mussels *Mytilus edulis*. (**a**) Large granulocytomas (*) located within the vesicular connective tissue (VCT). Haematoxylin and eosin (H&E) stain. (**b**) Large granulocytoma associated with haemocytic infiltration at the periphery of the lesion (*). H&E stain. (**c**) Detail of eosinophilic granulocytes (arrows) within the lesion. H&E stain. (**d**) Inclusions containing basophilic prokaryotic organisms (arrows) in the epithelium of gill lamellae (arrows). H&E stain.

Two surveys of blue mussels *Mytilus edulis* at Cremyll Ferry on the Tamar estuary conducted on 8 June and 21 July 2013 showed granulocytomas in 6.7% and 7.1%, respectively, of the mussels sampled (Table 1). Histopathological changes consisted of large granulocytomas in the vesicular connective tissue (VCT) associated with prominent haemocytic infiltration at the periphery of the lesion (Figure 1a–c). Examination of Gram-stained tissue sections did not reveal the presence of bacteria within the lesions. Less commonly observed were intracellular inclusions containing basophilic microcolonies of bacteria in the digestive cells of the digestive tubules (in 2% of the samples collected in June) (Supplementary Material Figure S1). The severity of the lesions in the affected specimens was noted as severe based on the degree of host inflammation and the occurrence of large granulocytomas.

A nearby site (Jupiter Point) of the same estuarine system was sampled in 2016, 2018, and 2019. All the specimens were sampled in summer months, except for the sampling conducted in 2019, which was performed in winter (Table 1). The pathology features observed across the years were consistent with those described in 2013, with the presence of the bacteria associated with severe inflammatory reactions and the formation of granulocytomas. Prokaryotic inclusion bodies in the digestive tubules were not observed. In samples collected in 2016, in addition to typical granulocytomas, basophilic prokaryotic inclusion bodies were also observed within the gill epithelium in a small proportion of specimens (5%) with no apparent inflammatory response (Figure 1d). The number of specimens exhibiting granulocytomas increased over the years, from 7% in 2013 to 17.8% in 2019.

### 2.2. Nanopore Sequencing of the 16S rRNA Gene of Francisella Halioticida Infecting Blue Mussels

Four specimens (samples RA13082 no. 26 and 154, and RA13085 no. 66 and 134) showing granulocytomas and haemocytic infiltration in the VCT were selected for *16S rRNA* gene Nanopore sequencing using 16S universal primers. Nucleotide consensus sequences of 1449–1450 bp were obtained. The four specimens analysed showed nucleotide sequence similarity of 99.9–100% among them and 99.8–100% with the closest related organism *F. halioticida* (Table 2).

A fragment of the *16S rRNA* gene with 423 bp of *F. halioticida* was Sanger sequenced to assess putative sequencing errors of the consensus obtained by Nanopore sequencing (Supplement Figure S2). The overlapping region of the sequences obtained by Sanger and Nanopore sequencing were identical.

**Figure 2:**
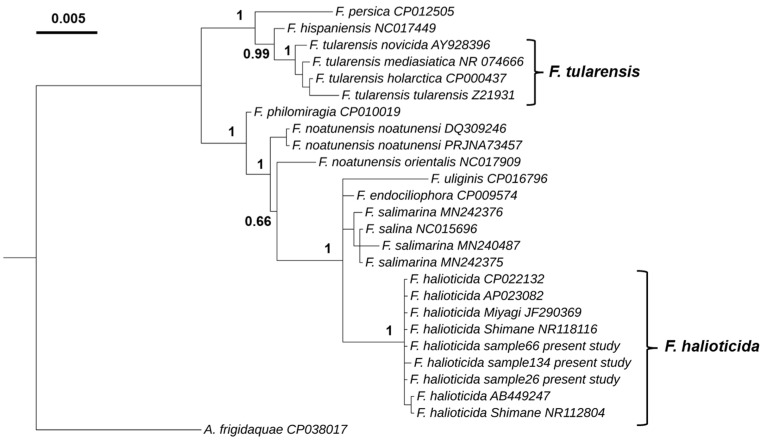
Bayesian phylogenetic tree based on 16S rRNA gene sequences (1449 nt) of species of the genus *Francisella*. The 16S rRNA gene of *Allofrancisella frigidaquae* strain SYSU 10HL1970 (GenBank accession number CP038017) was used as an outgroup. Node labels show posterior probabilities.

Our phylogenetic analysis using the *16S rRNA* gene identified two major genetic lineages (Figure 2), with one clade comprising *F. tularensis*, *F. hispaniensis* and *F. persica*, and the other clade containing *F. philomiragia*, *F. noatunensis*, *F. uliginis*, *F. endociliophora*, *F. salina*, *F. salimarina* and *F. halioticida*. The *F. halioticida* infecting blue mussels in the UK is placed within the same cluster as isolates of *F. halioticida* previously described infecting abalone species. This cluster is only composed of *F. halioticida* isolates with a posterior probability of 1. The closest species to *F. halioticida* cluster were *F. marina*, *F. salimarina* and *F._endociliophora* with a nucleotide sequence identity of 98.9%.

### 2.3. Confirmation of Francisella Halioticida in the Lesions

**Figure 3:**
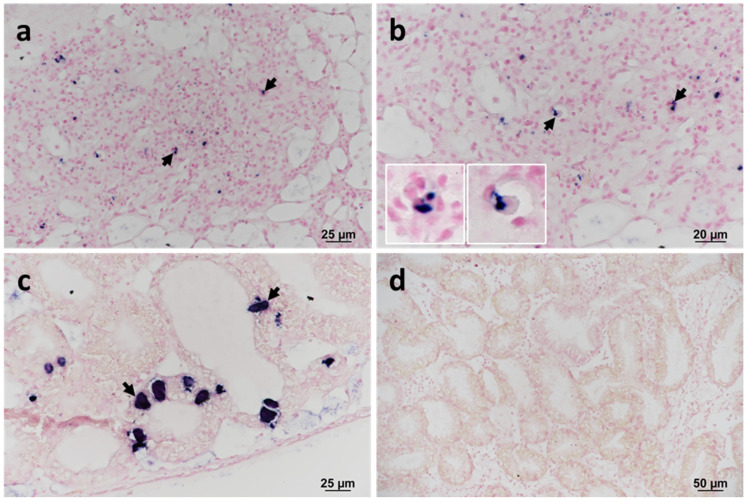
In situ hybridisation (ISH) of the *16S rRNA* gene of *Francisella halioticida* infecting blue mussels collected in 2013. The labelling is observed microscopically as dark blue staining. (**a**,**b**) Haemocytic infiltration within the vesicular connective tissue (VCT). (**b**) Insets show the detail of positively labelled granulocytes (arrows). (**c**) Positive labelling of intracellular prokaryotic inclusion bodies (arrows) within digestive cells of the digestive tubule epithelium. Note the absence of any associated inflammatory response. (**d**) Negative control using a non-specific probe.

The presence of *F. halioticida* DNA in blue mussels was confirmed by in situ hybridisation (ISH) in samples confirmed positive by sequencing (RA13028 no. 26 and 152). The labelling was observed in granulocytes located within areas of haemocytic infiltration of the VCT (Figure 3a,b). Prokaryotic inclusions in the digestive tubules of samples collected in 2013 also showed strong labelling (Figure 3c). No labelling was observed in infected mussels without a specific probe (Figure 3d).

Specimens showing granulocytomas from different samplings were selected and the presence of *F. halioticida* DNA was also confirmed by nested-PCR (Supplement Figure S2).

## 3. Discussion

This is the first report confirming the presence of the pathogen *F. halioticida* in a wild population of *M. edulis* in the United Kingdom. The presence of IMCs, traditionally noted as *Rickettsia*-like organisms (RLOs) or *Chlamydia*-like organisms (CLO), has been historically reported in mussels in the Tamar estuary [1], although these reports were based exclusively on histopathological descriptions. In a recent study using a 16S high-throughput amplicon sequencing approach, an unusually high proportion of reads (up to 67%) of a ~150 bp fragment showed 100% similarity with *Francisella philomiragia* strain ATCC 25,015 (AY928394.1) and *F. halioticida* strain Shimane-1 (AB449247). However, identification at the species level was not possible using short read lengths [3].

In the present study, we used nanopore technology to sequence a 1449 bp fragment of the *16S rRNA* gene of a bacterium infecting *M. edulis*. The sequences obtained showed a nucleotide sequence identity between 99.93 and 100% with published sequences of *F. halioticida* strains infecting abalone species [5,6].

Oxford Nanopore Technologies can sequence long reads, including amplicons with several thousand bases. Although the base-level accuracy of Nanopore raw reads is considerably lower than short-read sequencing platforms such as Illumina, high consensus-level sequencing accuracy can be achieved [15]. This was the case of the nanopore consensus sequences of *F. halioticida* obtained in the present study with a nucleotide sequence identity of 100% with the 423 bp fragment obtained by Sanger sequencing. However, an indel of one nucleotide was observed in a guanine homopolymer region in two of the consensus sequences obtained, which is the most commonly found error in Nanopore sequencing [16].

The presence of *F. halioticida* DNA within inflammatory lesions was confirmed by in situ hybridisation in selected specimens. Similar to the francisellosis reported in mussels in France [10], labelling was observed in granulocytes within large granulocytoma. Labelled granulocytes were also observed in areas of haemocytic filtration of the VCT as described in infected Yesso scallops [7]. Although positive labelling of prokaryotic inclusions was noted in the epithelium of the digestive tubules in some samples collected in 2013, their presence was not observed in other years, suggesting either an atypical presentation of *F. halioticida* or a co-infection with other inclusion forming bacteria. Although the description of francisellosis in abalone included a high number of bacteria-like particles observed in cells presumed to be phagocytes in a gill filament [5], prokaryotic cysts in the gills were not associated with an inflammatory response in the present study. Moreover, it remains unknown whether the bacterium causing the gill inclusions of blue mussels is in fact *F. halioticida* as the tissues sequenced were exclusively from the digestive gland.

In the present study, a *16S rRNA* gene probe was used for the ISH studies. Its cross-reaction with other bacteria in the tissues cannot be ruled out, particularly with the prokaryotic inclusions observed in the digestive tubules. In Yesso scallops, a strong cross-reaction with prokaryotic inclusions in the gills were noted when using a specific probe for *F. halioticida* [7]. Recent studies showed a predominant abundance of *Endozoicomonas* sp. organisms causing inclusions in gill and digestive gland tubules of various mollusc species, in general, associated with low inflammation and mild pathology [3,4]. Further studies are therefore required to fully understand the pathogenesis of *F. halioticida* in mussels and its interaction with other microbiota in the infected specimens. The specificity of probes for ISH studies could be improved by the use of specific oligonucleotide probes and in silico testing of specificity using software tools such as probeCheck and SILVA [17,18].

In mussels, two populations of haemocytes have been described: agranulocytes (hyalinocytes) and granulocytes (both acidophilic and basophilic). The granulocytes, in particular acidophilic granulocytes, have phagocytic activity [19]. The fact that a high number of eosinophilic (acidophilic) granulocytes were forming granulocytoma suggests a strong host immune response to *F. halioticida*. Mussel haemocytes can also generate a typical respiratory burst in defence of pathogens [20]. Other *Francisella* species, including *F. tularensis*, survive and replicate in phagocytic cells such as macrophages by regulating oxidative stress responses produced by the infected macrophages to promote resistance against reactive oxidative species (ROS) which contribute to its intracellular survival [21]. The survival mechanisms of *F. halioticida* in mussel granulocytes require further investigation, as well as the presence of transcriptional regulators of oxidative stress such as OyxR and SoxR homologs in the bacterium genome [22], which could be used as virulence markers for future studies.

After the confirmation of *F. halioticida* infection in samples collected in 2013, archived samples from 2016 to 2019 were scrutinized for the presence of granulocytomas typical of francisellosis. Histological examination showed similar lesions in the affected population over the years, with an increased prevalence from 7% in 2013 to 17% in 2019, the latest year surveyed. A similar scenario was described in France, where records related to the presence of numerous inflammatory granulocytoma in French mussels were noted from 2015 to 2020, with some of those records linked with mortality [10,23,24,25]. However, the mortality rates of mussels in France associated with *F. halioticida* were estimated at 20 and 56.5% [10], much lower than in abalone species (giant abalone, Japanese black abalone, and disk abalone) and Yesso scallops, where the bacteria infection was associated with 100% mortality [5,9,11]. In mussels, therefore, despite the presence of *F. halioticida* DNA in some lesions being confirmed ([10], this study), its pathogenicity still requires the confirmation of Koch’s postulates. Previous studies successfully isolated the bacterium from the haemolymph of infected abalone and cultured it in vitro using a modified Eugon agar supplemented with 1% haemoglobin in 70% seawater at 15 °C [5,6]. Unfortunately, the archived samples analysed in the present study did not include bacteriology analysis. A dedicated sampling is planned for 2022 to isolate *F. halioticida* from blue mussels from the Tamar estuary. If successful, the bacterium strain will be used for experimental challenges and whole-genome sequencing.

Whether the *F. halioticida* found in the United Kingdom is genetically identical to that infecting mussels in France or the Shimane and Miyagi isolates is currently unknown. A recent study showed differences in the genome of *F. halioticida* strains isolated from Yesso scallop and giant abalone in Japan, with an overall similarity of the whole genome sequence of 99.84%, and a lack of prolyl aminopeptidase activity in the strain isolated from Yesso scallop [11]. Although there is no sequence data available for the *16S rRNA* gene of the bacterium infecting mussels in France, the authors provided sequencing data for the housekeeping gene *DNA-directed RNA polymerase* beta subunit (*rpoB*), which showed a similarity of 99.9% with the giant abalone strain [10]. Taken all together, the bacterium infecting mussels might represent a different strain to the giant abalone and the Yesso scallop strains, however, the sequencing of the genome of *F. halioticida* infecting mussels is required to test this hypothesis further.

Since its first detection in Japan in 2005 [5], *F. halioticida* has been observed associated with mortalities in abalone hosts in Japan, Yesso scallops in Japan and Canada, and more recently, mussels in France [5,7,8,10]. However, the current impact of this infection in worldwide mariculture is unknown, and studies on potential hosts or reservoirs are scarce.

The family *Francisellaceae* also comprises pathogens of fish. Pathogenic bacteria in teleost fish have been classified either within the *F. piscicida* branch, or closely related to *F. noatunensis* subsp. *orientalis*. These pathogenic bacteria have been isolated worldwide from a variety of fish species such as Atlantic cod (*Gadus morhua* L.), tilapia (*Oreochromis* spp.), and Atlantic salmon (*Salmo salar* L.), among others [26]. Similar to the pathology observed in molluscs, infection with species of the genus *Francisella* cause high mortalities in fish associated with granulomatous inflammatory reactions [26]. The pathogenicity of *F. halioticida* in teleost fish was tested in red sea bream (*Pagrus major* Temminck Schlegel). No mortality was observed either by injection or immersion routes of infection [5], suggesting that *F. halioticida* is a low risk for finfish farming. In addition, the threat to humans from the fish and shellfish pathogenic *Francisella* species is considered very low [26]. However, the risk that *F. halioticida* might pose to other important shellfish species cultured in Europe, as Pacific oysters (*Crassostrea gigas*, Thunberg), requires further investigation. Moreover, recent reports of *F. halioticida* in mussels in Brittany and Normandy, France [10] and southwest England (this study) suggest a broad geographical distribution of this bacterium, at least already established in coastal zones around the English channel.

In summary, in the present study, we report the presence of *F. halioticida* in a wild population of blue mussels, first detected in archived samples collected in 2013 and still present in the population in 2019. However, it could be possible that this infection was present before the first sampling conducted in 2013. Despite the persistence of the infection within the population over the years, the number of animals showing severe pathology associated with granulocytoma was considered low. Diagnostic tests for surveillance are required to identify the bacterium in specimens showing mild pathology and asymptomatic. Due to the lack of pathogenicity studies, it is premature to evaluate how pathogenic this infection is to blue mussels and whether mussels are less susceptible to suffer mortalities than abalone species and Yesso scallop. Environmental factors might also contribute to the severity of the lesions [3].

## 4. Materials and Methods

### 4.1. Mussel Sampling in Tamar Estuary

An unusually high number of empty shells and population declines of blue mussels in the Tamar estuary have been sporadically noted in the last decade. Two dedicated health check surveys to determine possible reasons for this decline were carried out in 2013, and then in 2016, 2018, and 2019. The number of sampled mussels per year is shown in Table 1. In 2013, mussels were collected from Cremyll Ferry, Devon (50°21′ 38.34′′ N, 4°10′32.36′′ W), near the River Tamar estuary mouth, and in later years (2016 onwards) from Jupiter Point (50°23′25.50′′ N, 4°13′48.95′′ W), on the River Lynher as it joins the Tamar estuary.

Tissues were sampled for histology and molecular analysis. For all collection years, samples were processed for histological analysis and sections stained with haematoxylin and eosin (H&E) following standard protocols [27]. Giemsa and Gram stains were also used for the elucidation of bacteria in tissues [27]. Tissue sections were examined with a Nikon Eclipse E800 microscope (Nikon, Gillingham, UK) with images captured using Nis-Elements imaging software (Nikon, Gillingham, UK).

For molecular analysis, in 2013 sections of the digestive gland were dissected, preserved, and processed separately. Samples from June were preserved in 100% molecular-grade ethanol, and stored at −20 °C. Samples from July were flash-frozen in liquid nitrogen and stored at −80 °C before being thawed in RNALater (Qiagen, Germantown, MD, USA). All DNA extractions from 2013 were carried out using the DNeasy Blood and Tissue Kit (Qiagen, USA) with the standard kit protocol. Samples from subsequent years were collected as steaks comprising all tissue types and were preserved in 100% ethanol and stored at −20 °C. DNA was extracted using a phenol:chloroform protocol [28].

### 4.2. 16S rRNA Gene Nanopore Sequencing

#### 4.2.1. Library Preparation and Sequencing

Four specimens of *M. edulis* sampled in 2013 showing granulocytomas and haemocytic infiltration in the VCT were selected for Nanopore sequencing analysis. The universal primer pair FD1 (5′-TTTCTGTTGGTGCTGATATTGCAGAGTTTGATCCTGGCTCAG-3′) and rP2 (5′-ACTTGCCTGTCGCTCTATCTTCACGGCTACCTTGTTACGACTT-3′) described by [29] but tailed (underline nucleotides) to allow incorporation of Oxford Nanopore barcode sequences was used to amplify the nearly full-length 16S ribosomal DNA (rDNA) (approximately 1500 bp) from DNA extracted from digestive gland tissue. PCR reactions were performed in a final reaction volume of 50 µL and contained 10 µL of 5X Colourless GoTaq Flexi Buffer (Promega, Southampton, UK), 30 µL of nuclease-free water, 1.25 µL of dNTP (10 mM, New England Biolabs, Hitchin, UK), 5 µL of MgCl2 (25 mM) (Promega, Southampton, UK), 0.5 µL of each primer (10 µM), 0.25 mL of GoTaq G2 Hot Start (Promega, Southampton, UK) and 2.5 µL of template DNA. The amplification cycle consisted of an initial denaturation step at 94 °C for 5 min, followed by 35 cycles of 1 min at 94 °C, 1 min at 55 °C, 1 min at 72 °C, followed by a final extension of 10 min at 72 °C. The PCR reactions were performed in a Master Cycler Nexus X2 thermocycler (Eppendorf). The amplicons were then cleaned up using AMPure XP beads (New England Biolabs, Hitchin, UK) at a 1:1 ratio and quantified using the Qubit DNA HS Assay (Invitrogen, Life Technologies, Paisley, UK). The PCR Barcoding Expansion Pack 1-96 (EXP-PBC096) and the Ligation sequencing kit (SQK-LSK109) were used to prepare the sequencing library as described by Oxford Nanopore Technology (ONT).

Seventy-five microlitres of the library was then loaded to a FLO-MIN106 R9.4.1 flow cell that had been previously primed. The flow cell was operated using MinKNOW and ran until it obtained at least 15,000 reads per sample.

#### 4.2.2. Bioinformatic Analysis

Raw sequence data were base-called using Guppy v4.5.2 (ONT, Oxford, UK) using the high accuracy base-calling model dna_r9.4.1_450bps_hac. The sequence data were demultiplexed using Guppy v4.5.2. and chimaeras and adaptors were removed using Porechop (v0.2.3, https://github.com/rrwick/Porechop accessed on 20 October 2017). Run metrics were visualized using the Nanoplot (v1.20.0), and reads were filtered (-q 10) using Nanofilt (v2.3.0). Cutadapt (version 3.2) was then used to remove the annealing regions (using -j 20 and -e 0.20) as well as reads with less than 1200 bases (--minimum-length 1200).

### 4.3. Francisella Halioticida PCR and Sanger Sequencing

A fragment of 423 bp of the *F. halioticida 16S rRNA* gene was amplified by nested-PCR using the previously published primers FD1 and rP2 [30] described above and the set of primers Megai-60 (5′-CGGTAACAGGAGAAGCTTGCTTCT-3′) and Megai-480r (5′-TCTTTGGGTAACGTCCTTCTCATG-3′) [5]. Both rounds of PCR reactions were performed in a 50 µL reaction volume consisting of 1× green GoTaq^®^ Flexi buffer, 2.5 mM MgCl2, 1 mM dNTP mix, 1.25 units of GoTaq^®^ G2 Hot Start Polymerase (Promega, Southampton, UK), 50 pmol of each primer and 2.5 µL of digestive gland DNA. After an initial denaturing step of 5 min at 95 °C, samples were subjected to 30 cycles of 30 s at 95 °C, 30 s at 55 °C, 1 min at 72 °C, followed by a final extension step of 10 min at 72 °C in a Mastercycler nexus X2 (Eppendorf, Stevenage, UK).

Both strands of the nested PCR product were Sanger-sequenced using an ABI Prism Dye Terminator cycle sequencing kit (PerkinElmer, Buckinghamshire, UK) following the manufacturer’s protocol. The nucleotide similarity of the consensus sequence with the primers sequence removed (375 bp) was determined by BLASTn (NCBI nucleotide database assessed in May 2021) [31].

### 4.4. Phylogeny Studies

An alignment of 16S rRNA gene sequences (1454 nucleotides) was carried out with MAFFT (https://www.ebi.ac.uk/Tools/msa/mafft/, accessed on 7 January 2022) using sequences available in GenBank from *F. persica* (CP012505), *F. hispaniensis* (NC017449), *F. tularensis* (AY928396, NR074666, CP000437, Z21931), *F. philomiragia* (CP010019), *F. noatunensis* (DQ309246, PRJNA73457, NC017909), *F. uliginis* (CP016796), *F. endociliophora* (CP009574), *F. salimarina* (MN242376, MN240487, MN242375), *F. salina* (NC015696) and *F. halioticida* (CP022132, AP023082, JF290369, NR118116, AB449247, NR112804) and the ones obtained in the present study. The Bayesian analysis was conducted using the MrBayes-3.2.7 software [32]. The GTR substitution model was used with gamma-distributed rate variation across sites and a proportion of invariable sites (GTR + I + G). An initial Markov chain Monte Carlo run with 20,000 generations was performed followed by a second run with 100,000 generations that resulted in a standard deviation of split frequencies below 0.01. The interactive tree of life (iTOL) webserver (https://itol.embl.de, accessed on 7 January 2022; [33]) was used to display the phylogenetic tree.

### 4.5. In Situ Hybridisation

Embedded tissues in paraffin blocks were sectioned and placed on silane-treated slides (Merck Life Science, Gillingham, UK) for in situ hybridisation (ISH) analysis. A digoxigenin (DIG)-labelled DNA probe of 423 bp of the *F. halioticida 16S rRNA* gene was generated by PCR using DIG-labelled dNTPs (Merck Life Science, Gillingham, UK) and the primers Megai-60 and Megai-480r as described above. A non-specific probe was designed to label the 16S rRNA gene of *Endozoicomonas*-like organisms infecting king scallops as described by Cano et al. [4].

ISH assays were carried out following standard protocols [30]. Briefly, tissues were permeabilized with Proteinase K (100 µg mL^−1^) (Promega, Southampton, UK) for 30 min at 37 °C in a moisture chamber. The DIG-labelled probe was diluted 1:10 in hybridisation buffer (60% formamide, 10% dextran sulfate, 2× saline sodium citrate buffer (SSC), and 0.2 μg μL^−1^ salmon sperm DNA) and denatured at 95 °C for 5 min before hybridisation overnight at 42 °C. Two post-hybridisation washes containing 2× SSC, 6 M urea, and 0.2% bovine serum albumin (BSA) were performed at 42 °C for 15 min each. Tissue sections were blocked with 6% skimmed milk (Merck Life Science, Gillingham, UK) and incubated with an anti-DIG monoclonal antibody conjugated to alkaline phosphatase (Merck Life Science, Gillingham, UK) for 1 h. The hybridisation signal was revealed using NBT/BCIP (Merck Life Science, Gillingham, UK) and nuclei counterstained using nuclear fast red stain (Merck Life Science, Gillingham, UK).

## Figures and Tables

**Table 1:** Prevalence of granulocytomas in the vesicular connective tissue (VCT) of blue mussels (*Mytilus edulis*) sampled in the Tamar estuary. No: number of individuals analyzed.

Date_ID	Sampling Site	No	Granulocytomas (%)
08/06/2013_RA13082	Cremyll Ferry	162	6.7
21/07/2013_RA13085	Cremyll Ferry	153	7.1
19/06/2016_RA16043	Jupiter Point	120	17.5
13/08/2018_RA18075	Jupiter Point	57	15.7
21/02/2019_RA19012	Jupiter Point	56	17.8

**Table 2:** Percentage of nucleotide identity of the *16S rRNA* gene of *Francisella halioticida* infecting blue mussels (this study) with published sequences of *F. halioticida* and close relatives within the *Francisella* taxa. The entire table can be found in Supplement Table S1.

Bacterium Strain	*F. halioticida* in Mussels	Host	GenBank Acc.	Reference
*F. halioticida* DSM 23729	99.93%	Giant abalone	CP022132.1	Unpublished
*F. halioticida* Miyagi-1	99.93%	Disk abalone	JF290369.1	[6]
*F. halioticida* UTH170823	99.93%	Yesso scallop	AP023084.1	[11]
*F. halioticida* Shimane-1	99.86%	Giant abalone	NR_112804.1 AB449247.1	[5]
*Francisella* sp. FSC1006	98.90%	Marine Ciliate *Euplotes raikovi*	CP009574.1	[12]
*F. marina* E-103-15	98.83%	Spotted rose snapper *Lutjanus guttatus*	MH057676.1	[13]
*F. philomiragia* GA01-2794	97.81%	Human *Homo sapiens*	CP009440.1	[14]

## Data Availability

The consensus sequence of a fragment of 1450 bp of the *16S rRNA* gene of the *F. halioticida* infecting blue mussels obtained by Nanopore sequencing was deposited on the NCBI database with accession numbers SUB10902594 Seq2_Oxford OM142658; SUB10902594 Seq3_Oxford OM142659; SUB10902594 Seq4_Oxford OM142660; and SUB10902594 Seq5_Oxford OM142661. The consensus sequence of a fragment of 375 bp the *16S rRNA* gene of the *F. halioticida* infecting blue mussels obtained by Sanger-sequencing was deposited on the NCBI database with accession number SUB10902594 Seq1_Sanger OM142657. The slides from the pathology and in situ hybridisation analysis are available upon request.

## Supplementary Materials

The following are available online at https://www.mdpi.com/article/10.3390/pathogens11030329/s1, Figure S1: Other pathogens identified in the sampled mussels. (a) Mytilicola-like sp. (asterisk) in the stomach lumen. (b) Co-infection of *Marteilia pararefringens* and prokaryotic cysts in the digestive tubules. Black arrows: eosinophilic sporocysts; blue arrows: prokaryotic cyst, Figure S2. Nested-PCR amplification of a fragment of 423 bp of the 16S rRNA gene of *Francisella halioticida* in *Mytilus edulis* using the set of primers FD1/rP2 (Weisburg et al., 1991) for the first round of PCR; and Megai-60/Megai-480r (Kamaishi et al., 2010) for the second round of PCR. The agarose gel shows the amplified fragment of 423 bp in positive samples (asterisks). Water was used as negative control (W). Additionally, 650 ng of a 100 bp DNA ladder (Promega) was used as a molecular weight marker (L), Table S1: Percentage of nucleotide identity of the 16S rRNA gene of *Francisella halioticida* infecting blue mussels (this study) with published sequences of *F. halioticida* and close relatives within the *Francisella* genus.
