# Supplementary material for: First Detection of Francisella halioticida Infecting a Wild Population of Blue Mussels Mytilus edulis in the United Kingdom
Source: Pathogens. 2022 Mar 8;11(3):329. doi: 10.3390/pathogens11030329 (PMC8953295; doi:10.3390/pathogens11030329)
Supplement: Supplementary file 1 [file pathogens-11-00329-s001.zip › pathogens-1568745-supplementary.pdf]

pathogens

MDPI

Supplement

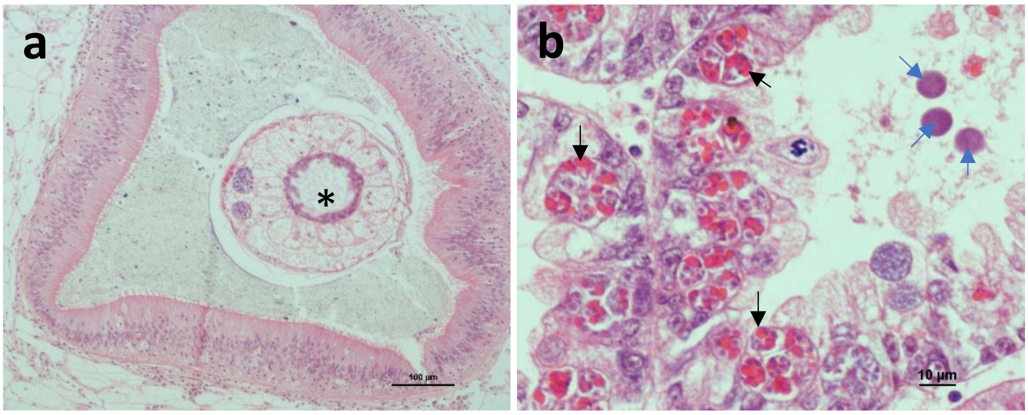

Figure S1. Other pathogens identified in the sampled mussels. (a) *Mytilicola*-like sp. (asterisk) in the stomach lumen. (b) Co-infection of *Marteilia pararefringens* and prokaryotic cysts in the digestive tubules. Black arrows: eosinophilic sporocysts; blue arrows: prokaryotic cysts.

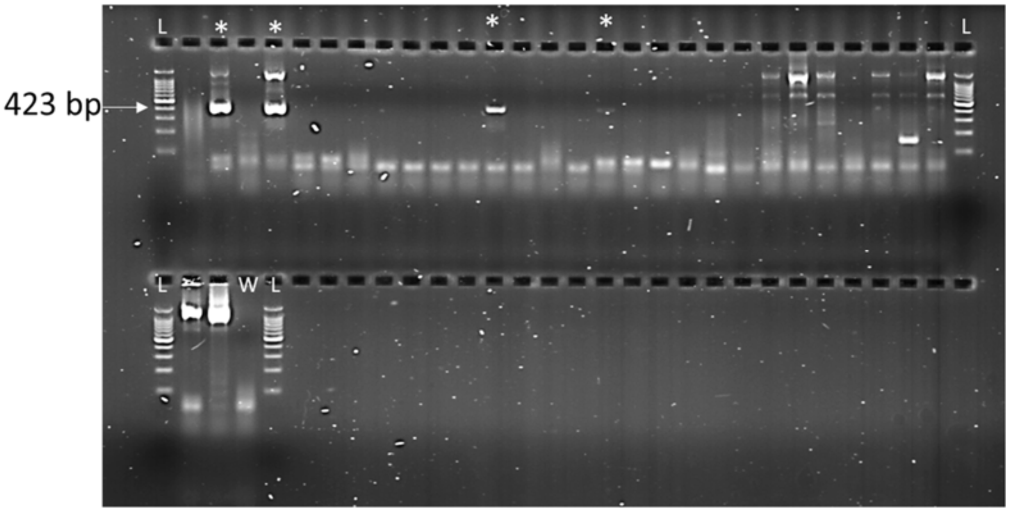

Figure S2. Nested-PCR amplification of a fragment of 423 bp of the 16S rRNA gene of *Francisella halioticida* in *Mytilus edulis* using the set of primers FD1/rP2 (Weisburg et al., 1991) for the first round of PCR; and Megai-60/Megai-480r (Kamaishi et al., 2010) for the second round of PCR. The agarose gel shows the amplified fragment of 423 bp in positive samples (asterisks). Water was used as negative control (W). 650 ng of a 100 bp DNA ladder (Promega) was used as a molecular weight marker (L).

Supplement Table S1. Percentage of nucleotide identity of the 16S rRNA gene of *Francisella halioticida* infecting blue mussels (this study) with published sequences of *F. halioticida* and close relatives within the Francisella taxa.

		1	2	3	4	5	6	7	8	9	10	11	12	13	14	15	16
1	*F._salina*_NC015696																
2	*F._noatunensis*_DQ309246	98.72															
3	*F._endociliophora*_CP009574	99.64	98.65														
4	*F._uliginis*_CP016796	98.58	97.79	98.65													
5	*F._tularensis*_Z21931	96.79	97.36	96.87	96.15												
6	*F._persica*_CP012505	97.29	97.44	97.22	96.58	97.93											
7	*F._hispaniensis*_NC017449	97.22	97.86	97.15	96.44	99.00	98.58										
8	*F._philomiragia*_CP010019	98.58	99.43	98.50	97.65	97.79	97.65	98.43									
9	***F._halioticida*_NR112804**	98.72	97.86	98.79	98.01	96.15	96.72	96.44	97.93								
10	***F._halioticida*_AB449247**	98.72	97.86	98.79	98.01	96.15	96.72	96.44	97.93	**100**							
11	***F._halioticida*_NR118116**	98.79	97.93	98.86	98.08	96.23	96.79	96.51	98.01	**99.93**	**99.93**						
12	***F._halioticida*_JF290369**	98.79	97.93	98.86	98.08	96.23	96.79	96.51	98.01	**99.93**	**99.93**	**100**					
13	***F._halioticida*_AP023082**	98.79	97.93	98.86	98.08	96.23	96.79	96.51	98.01	**99.93**	**99.93**	**100**	**100**				
14	***F._halioticida*_CP022132**	98.79	97.93	98.86	98.08	96.23	96.79	96.51	98.01	**99.93**	**99.93**	**100**	**100**	**100**			
15	***F._halioticida*_S26***	98.79	97.93	98.86	98.08	96.23	96.79	96.51	98.01	**99.93**	**99.93**	**100**	**100**	**100**	**100**		
16	***F._halioticida*_S134***	98.72	97.86	98.79	98.01	96.15	96.72	96.44	97.93	**99.86**	**99.86**	**99.93**	**99.93**	**99.93**	**99.93**	**99.93**	
17	***F._halioticida*_S66***	98.79	97.93	98.86	98.08	96.23	96.79	96.51	98.01	**99.93**	**99.93**	**100**	**100**	**100**	**100**	**100**	**99.93**
